# Royal Jelly Enhances the Social Status of Submissive Rats by Restoring Balance to the Disturbed Gut–Brain Communication

**DOI:** 10.3390/foods14050819

**Published:** 2025-02-27

**Authors:** Feng Zhu, Jinchun Xu, Tian Wang, Ruili Yang, Biao He, Hui-Li Wang, Yi Xu

**Affiliations:** School of Food and Biological Engineering, Hefei University of Technology, Hefei 230000, China; zf1764230916@163.com (F.Z.); a2241295211@163.com (J.X.); halotianwang@163.com (T.W.); young02202@outlook.com (R.Y.); 13085062468@163.com (B.H.)

**Keywords:** royal jelly, social rank, *Escherichia*, gut microbiota, gastrin-releasing peptide

## Abstract

Royal jelly (RJ) has long been considered a crucial dietary component in dictating caste differentiation in honeybees. As a nutritional additive, royal jelly imparts a broad range of benefits to mammals and humans; however, its precise impact on the social hierarchy of these advanced animals is not yet fully understood. This study aims to determine whether the benefits of royal jelly can be transferred to rats to alter their social ranks and uncover the underlying mechanisms. A submissive model was established by inducing dysbiosis in rats, via the persistent exposure of vancomycin. Royal jelly at a dose of 2.5 g/kg was daily administered to the subject rats during postnatal weeks (PNW) 6 and 7. At the end of the intervention, animals were subjected to agonistic, water and tube competition tests, in order to assess their dominance status. As revealed by the results, the RJ treatment significantly improved the social rank of the dysbiotic rats, demonstrating that RJ can elicit positive effect on the social behaviors (caused by dysbiosis) of rats. All behavioral paradigms yielded consistent results, with no notable differences in body weight or anxiety levels. Regarding gut microbiome, vancomycin exposure caused the dysbiosis of the subject rats, which was partially reversed by treatment with royal jelly. Specifically, the intestinal presence of Proteobacteria was profoundly attenuated by the RJ supplementation, resulting in a comparable level with the intact/dominant rats. At the genus level, both *Escherichia* and *Clostridium* displayed similar dynamics in relation to Proteobacteria, implying their involvement with the RJ-mediated dominance switching. Transcriptomic analysis in the medial prefrontal context showed that the expression of a broad range of genes was influenced by RJ intake, embodying various pathways related to neuronal transmission such as neuroactive ligan–receptor interaction, the synaptic vesicle cycle, etc. By virtue of correlation analysis, *Escherichia*, *Akkermansia* and *Clostridium* were strongly associated with a set of gene modules around gastrin releasing peptide (*Grp*) and signaling pathways around *Rps6ka3*, establishing an intrinsic gut–brain communication. Furthermore, the infection trials of *Escherichia* significantly degraded the social ranks of the RJ-remedied rats in tube tests, while a series of cerebral genes like *Grpr* and *Grpel1*, as well as prefrontal spine density, were concordantly altered, underscoring the critical role of the gut–brain link in deciding the outcomes of the dyadic contests. In summary, this is an intriguing example of how royal jelly can influence the social ranks of mammals, emphasizing the importance of microbe–host interaction in mediating this species-spanning function of royal jelly in shaping social hierarchy.

## 1. Introduction

Royal jelly (RJ) is a honeybee (*Apis mellifera*) secretion that is used as the major nutritional factor dictating caste differentiation among honeybees, that is, driving adult females to form two independent castes, the queen and the worker [1,2,3,4,5]. In the fields of food nutrition, RJ serves as an excellent example of how nutrition can influence developmental outcomes [6]. The major royal jelly proteins (MRJPs) in royal jelly are regarded as the key component in influencing the division of labor among honeybee workers [7]. However, this conclusion is seriously challenged by other researchers, who believe that MRJPs are not the single key for queen caste determination [8]. Despite conflicting data, it is well established that caste is nutritionally regulated, and only larvae fed exclusively on royal jelly can develop into queens [8,9]. Nonetheless, it has never been claimed that royal jelly could influence the social hierarchy in mammals or humans. However, based on the intrinsic activity of RJ in modulating social hierarchy in honeybees, the present study raised the hypothesis that similar activity might be observed in mammals.

RJ has been applied in the human body as a food additive, showing a diverse range of nutritional merits, as exemplified by improved reproduction, as well as antioxidant, anti-inflammatory, anti-cancer and antibacterial effects [10,11,12,13]. In terms of neuroregulatory activity, RJ was found potent in promoting neurogenesis by treating neural stem cells, alleviating memory impairment of Alzheimer’s disease and antiaging, among other benefits [14,15,16]. Notably, this effect is likely to arise from the modulatory activity of RJ towards gut microbiota, as even microbiota in the healthy mice was prone to RJ intervention [2]. Despite these advancements in understanding the brain-related activities, the relationship between RJ and social behaviors in higher organisms remains unclear.

A strong multidimensional link exists between mammalian nutrition and social behavior. A maternal high-fat diet is generally regarded as a risk factor for social deficits associated with autism spectrum disorder [17]. In this context, the colonization of a single commensal strain is sufficient in correcting oxytocin levels, long-term potentiation and social deficits in the offspring, which is valid proof that nutrition can pose beneficial or adverse effect on social behaviors. Our prior study [18] also demonstrated that the use of probiotics is competent in rescuing the social defeats induced by gut dysbiosis.

The microbiota–gut–brain axis (MGBA) is a complex two-way communication system involving complex interactions between the gut microbiota and the central nervous system (CNS), which is regularly mediated by immune factors, endocrine factors, metabolites and vagus nerves. [19] This axis regulates the host’s physiological function, mood, cognition and behavior through neural, endocrine, immune and metabolic pathways [20]. Studies in germ-free animals have shown that early gut flora deficiency leads to abnormal blood–brain barrier permeability, microglia dysfunction and social behavioral deficits, which is a direct piece of evidence of a connection between gut microbiota and social behaviors [17]. In our previous study [18], a gut–brain route with a focus at butyrate and histone acetylation was proposed to play crucial roles in the social subordination caused by dysbiosis. Therefore, social behaviors are profoundly modulated by the microbiota–gut–brain axis under various physiological conditions.

Social rank is a universal hierarchical behavior among grouped animals ranging from insects to primates [21,22,23,24]. Through competitive encounters, the organisms can be divided into the dominant and subordinate/submissive subsets, which further determine the individual distribution of resources, reproduction and territory, thereby impacting their survival and mental health [18,25,26]. In our previous study, social dominance was surprisingly predetermined by gut microbiota, whereas dysbiosis led to an extremely submissive state during dyadic contests [18]. Given this knowledge, it then raises the question if this adversity can be counteracted via rebiosis mediated by nutrition, such as by royal jelly. Besides, social hierarchy is routinely manifested by multiple behavioral paradigms such as water competition, agonistics, tube tests, etc. [22], which are precisely adopted in the current study to link them with RJ-mediated remediation. Our previous study [18] demonstrated the validity of using antibiotic-induced dysbiosis to establish the social defeat model. While it is not the classic approach, this treatment was literally linked with the microbiota alterations, which is a major topic of this study. Furthermore, the trials in our group discovered that this paradigm could be simplified by using vancomycin alone, an approach then adopted here in a practical sense.

To this end, this study aims to investigate the caste-modulatory effect of RJ in rats, along with the intermediary pathways involved. To achieve this, the antibiotic-exposed paradigm was used to establish a socially submissive model, which was then employed to evaluate the restorative activity of RJ. Additionally, the gut–brain pathway was delineated using multi-omics approaches, while the roles of specific microbes and their correlations with the expression of neuron-related genes were further clarified. This study provides a new example of transferring the caste-switching capability of royal jelly to mammals, shedding light on the novel aspects of nutritional influences on social ranks.

## 2. Materials and Methods

### 2.1. Chemical and Drugs

Vancomycin hydrochloride was purchased from Bio Basic Inc. (Shanghai, China). Royal jelly was purchased from Nanjing Laoshan Pharmaceutical Co. (Nanjing, China). Regarding ingredients, RJ contains 1.84% 10-hydroxy-2-decenoic acid. RJ was stored at −20 °C until it was dissolved in saline and used in animal studies.

### 2.2. Bacterial Strains

*Escherichia coli* (*E. coli*) was stored in my lab and cultured in LB medium at 37 °C for 12 h. The culture was centrifuged at 8000× *g* for 5 min and suspended to obtain a suspension of 1 × 10^9^ CFU/mL for further usage. Male rats were supplemented with *E. coli* by gavage at a dosage of 1 × 10^9^ CFU/d/rat during PNW8-9. The control group was given saline by gavage.

### 2.3. Animals

Sprague Dawley (SD) rats were obtained from the Laboratory Animal Center of Anhui Medical University and were housed in specific pathogen free (SPF) conditions with a 12 h light/dark cycle. The rats were supplied with pelleted rodent food and water. Male rats were gavaged daily with vancomycin hydrochloride (100 mg/kg, dissolved in saline) at PNW4 to 5. At PNW6 to 7, royal jelly (2.5 g/kg, dissolved in saline) and saline were administered by daily gavage to two separate groups of rats, respectively. The treatment with royal jelly suspension and saline was discontinued one week before behavioral tests due to the adulthood requirement of behavioral testing, and feces were collected for the high-throughput sequencing at PNW8. The behavioral assessments were performed only when male rats reached adulthood (PNW9). All experimental procedures were approved and conducted by the Institutional Animal Care and Use Committee of Hefei University of Technology, China. The rats were randomly divided into separate groups without preferences, and rats with abnormal interactions like climbing frequently and those who were seriously ill were removed from the experiment to ensure that they would not influence the behavioral outcomes. All the experiments were grouped, performed and calculated by different people in a blind fashion to prevent experimental bias.

### 2.4. Agonistic Trial

The male rats were paired based on body weight (less than 10%), anxiety and locomotor ability. Animals were marked on their backs for identification, placed in neutral cages (46 cm × 31.5 cm × 20 cm) for 12 h and videotaped to document their behaviors. The agonistic assay was conducted from 9:00 to 21:00 every day, with the room temperature maintained at 26~27 °C and humidity at 45%~65%. The offensive behaviors were defined as keeping down, lateral threat, chasing and offensive upright, which were recorded by a video camera (Hikvision, Hangzhou, China) and quantified as dominant behaviors. The dominance index was calculated as a percentage of a competitor’s offensive behavior relative to the pair’s total score. The neutral cage between pairs was thoroughly cleaned to avoid olfactory cues. More than one paradigm was used here to characterize the social status due to the generally acceptable guidelines [22].

### 2.5. Open Field Test

The rats were placed in an open field (1 m × 1 m × 0.4 m) from the same position for 10 min of free movement, with the light intensity in the room maintained at a constant level. The central zone was defined as the central 9 cells, with a size of 20 × 20 cm and 25 cells. The total distance traveled and the time spent in the central area of the open field by the rats were recorded by a video camera and analyzed by ANY-maze software (version 7.45, Stoelting, Chicago, IL, USA). Anxiety levels were calculated as the time spent in the central area by each rat, divided by the total time.

### 2.6. Water Competition Test

The water competition device comprised a roofless chamber (60 cm × 60 cm × 60 cm), a waterspout and a camera. The waterspout was designed to allow only one rat to drink, and the camera was located above the opposite side of the water intake point to record the experiment. Before testing, all rats were caged in individual cages, and water consumption was recorded for three consecutive days. During the training and testing, rats were deprived of water for 22 h per day. Following this deprivation, each rat was trained to drink water alone for 3 min. After training, the rats returned to their original cages and were allowed to drink freely for 1 h. After five days of training, abnormal rats were excluded from competition testing during this period. On the sixth day, each rat was allowed to adaptively drink water for 10 s. Then two paired rats were placed in the device at the same time and competed for the waterspout. Each test experiment lasted for 3 min, and the behavioral tracks of the rats were videotaped. After training, the time each rat spent at the spout was counted as a measure of their respective dominance.

### 2.7. Tube Competition Test

The tube test was designed to measure the social hierarchy of the contesting duos. The test consists of three phases: habituation, training and testing. At the habituation stage, a short habituation tube was placed in the cage for 3 days to familiarize the rats with the environment. At the training stage, all rats went through the tube 10 times daily, 5 times from each side. The test was conducted for 2 days to familiarize the rats with the test procedure and environment. In the testing stage, a pair of rats were placed into both sides of the tube simultaneously. The recorded time began when they reached the middle of the tube and lasted until one rat was pushed out of the tube. The maximum duration of each test was 15 s. The actual duration (t_0_) for each test was then counted and converted to (2t_0_ − 15) for winners while maintaining actual values for losers, in order to exhibit comparisons. For dominance index, the winners using time less than “Mean − SEM” of all tests, within the range of “Mean ± SEM”, over “Mean + SEM” but less than 15 s were designated as 100, 75 and 60, respectively. The contestants that did not yield a result within 15 s were designated as 50.

### 2.8. 16S rRNA Sequencing and Analysis

The gut microbiota of rats in response to treatments was characterized using 16S rRNA sequencing. Prior to the behavioral trials, fresh feces from male rats were collected, homogenized and subjected to DNA extraction using the HiPure Stool DNA Kit B (Magen, Guangzhou, China). The extracted DNA was amplified by 16SV4 PCR, and a library was constructed using the Ion Plus Fragment Library Kit (Thermo Fisher Scientific, Beijing, China). Following the qPCR quantification, the pooled amplicons were subjected to sequencing using the IonS5TMXL (Thermo Fisher Scientific, Beijing, China). The reads were spliced using FLASHVersion 1.2.11, and the resulting spliced sequences served as the raw tag data. Subsequently, the raw tags were subjected to further processing through successive steps of data segmentation, filtration, chimera removal and OUT retrieval.

Data were analyzed using QIIME (Quantitative Insights into Microbial Ecology) (Version 1.9.1). The initial analysis of the differences in gut microbiota between the various groups was conducted using phylogenetically based unweighted UniFrac distances. Subsequently, alpha and beta diversity were analyzed using Perl. R software (Version 2.15.3) was used to conduct principal component analysis (PCA). Linear discriminant analysis (LDA) effect size (LEfSe) was analyzed with the default value of LDA score set as 4. All 16S rRNA datasets were uploaded to the NCBI Sequence Read Archive (accession numbers PRJNA1214425).

### 2.9. Transcriptomic Analysis and qPCR Validations

The total RNA was extracted from male rat mPFC (medial prefrontal cortex) tissues and used as a template for the synthesis of cDNA, as previously described [18]. The sequencing was carried out using the Illumina NovaSeq 6000 platform (San Diego, CA, USA). The significance of gene expression differences between groups was calculated by the differential expression analysis function in the DESeq2 R package, and significant genes with an FDR (false discovery rate) value of less than 0.05 were identified. KEGG (Kyoto Encyclopedia of Genes and Genomes) and GO (Gene Ontology) enrichment analyses were performed by Cluster Profiler R package. Gene co-expression networks were established using STRING (https://string-db.org/, accessed on 11 October 2024) and illustrated using Cytoscape (version 3.9.1, Seattle, WA, USA) based on gene co-expression matrices. Datasets are available through the GenBank databases (accession number PRJNA1214734). The expression levels of *Grp*, *Grpr*, *Grpel1*, *Rps6ka3* and other genes of interest were further examined by qPCR experiments using the primers listed in Table 1.

Mantel test was conducted as the multi-omics approaches of identifying the correlation variant between a specific bacterial taxon and the prefrontal expression levels of genes, among the studied treatments.

### 2.10. Golgi–Cox Staining

Golgi–Cox staining was used to visualize dendrites and dendritic spines in rat prefrontal cortex sections. Fresh rat brain tissues were placed in a light-avoiding incubation box containing Golgi staining solution and incubated in a constant temperature box at 37 °C for 48 h. After the incubation, the brain tissues were then sectioned with the size of 200 μm thickness. The sections were then subjected to a series of incubations and staining processes as described previously [27]. Subsequently, the stained mPFC pyramidal neurons were photographed and counted using MATLAB software (version 2022a, Natick, MA, USA).

### 2.11. Statistical Analysis

Statistical analyses of the data were performed with GraphPad Prism 8.0 and SPSS 21.0, and the data were shown as Mean ± SEM from at least three independent replicates. The significance of the differences between the paired experimental groups was analyzed using the paired *t*-test, while the significance of the differences between the unpaired experimental groups was analyzed using the unpaired *t*-test or one-way ANOVA if over three groups were involved. One-sample *t*-test was conducted to assess the dominance index against the reference value of 50. * *p* < 0.05, ** *p* < 0.01 and *** *p* < 0.001 were considered statistically significant.

## 3. Results

### 3.1. RJ Elevates the Social Rank of Submissive Rats

**Figure 1:**
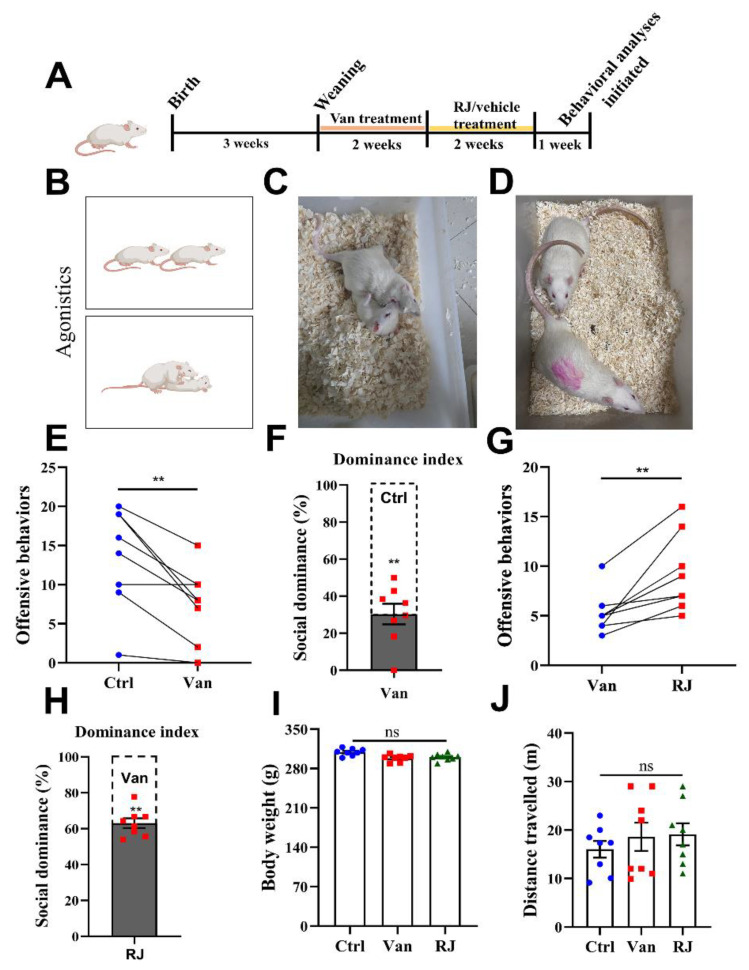
Agonistic behaviors in rats treated with vancomycin and royal jelly. (**A**) The experimental timeline of vancomycin (van) and royal jelly (RJ) treatment in rats. Vancomycin was gavaged to male rats at a dose of 100 mg/kg at PNW4 and 5, whereas royal jelly or saline was then added daily at a dose of 2.5 g/kg at PNW6 and 7. The behavioral test was initiated at PNW9 to evaluate the dominant status (*n* = 8). (**B**) The schematic illustration of the agonistic trials, wherein the duels occurred in a strange arena, and the agonistic actions were then recorded and counted. (**C**) A real-time representation of “clamping-down” behavior. (**D**) A real-time representation of “chasing” behavior. (**E**) The statistical comparisons of agonistic behaviors during the competitive encounters between untreated and van-treated groups (F(7,7) = 1.91). (**F**) The dominance index of the agonistic trials, which was calculated as the percentage of a specific contestant in the total behaviors arising from a specific duel. (**G**) Statistical comparisons of agonistic behaviors during the competitive encounters between van-treated and RJ-treated groups (F(7,7) = 3.41). (**H**) The dominance index of the agonistic trials between van-treated and RJ-treated groups. (**I**) Body weight measurements during the agonistic trials. (**J**) Distance travelled in the open-field test for rats with various treatments, which was then used as a parameter to represent their anxiety levels. Statistical analysis was performed using one-way ANOVA, paired *t*-test or one-sample *t*-test with the reference variable of 50. All data are expressed as mean ± SEM from three experiments. ** *p* < 0.01; ns, *p* > 0.05. Ctrl, untreated group; Van, vancomycin-treated group; RJ, royal jelly and vancomycin treated group.

As previously described, antibiotics can be used to induce gut dysbiosis, resulting in a socially submissive state [18]. Based on this, vancomycin was adopted here to expose SD (Sprague Dawley) rats from PNW4 to PNW5 (Figure 1A). Agonistic competition was then used to represent the social hierarchy of the tested rats. In this competitive encounter (Figure 1B), clamping-down (Figure 1C) and chasing (Figure 1D) behaviors were then recorded as the agonistic points derived by the dominant rats. As revealed by this paradigm, the introduction of vancomycin lowered the social rank of rats, since they were most frequently defeated in the agonistic trial when combating with the intact conspecifics (Figure 1E). In terms of dominance index, the controlled group gained an advantage of 63.08 ± 2.71 over their dysbiotic rivals (Figure 1F).

Subsequently, royal jelly (RJ) was orally gavaged to the submissive rats from PNW6 to PNW7 at a dosage of 2.5 g/kg, which was selected due to the reference with the previous literature [28]. Their efficacy was then assessed in the agonistic trials, whereas the representative agonistic behaviors were counted during this dyadic engagement. According to the results, RJ significantly elevated the social rank of the vancomycin-exposed rats, suggesting the profound restorative effect on this type of social behavior (caused by dysbiosis) (Figure 1G,H). Meanwhile, the tested rats did not differ in their body weight (Figure 1I) or anxiety levels (Figure 1J), a piece of evidence that this impact was solely imposed by the use of RJ.

**Figure 2:**
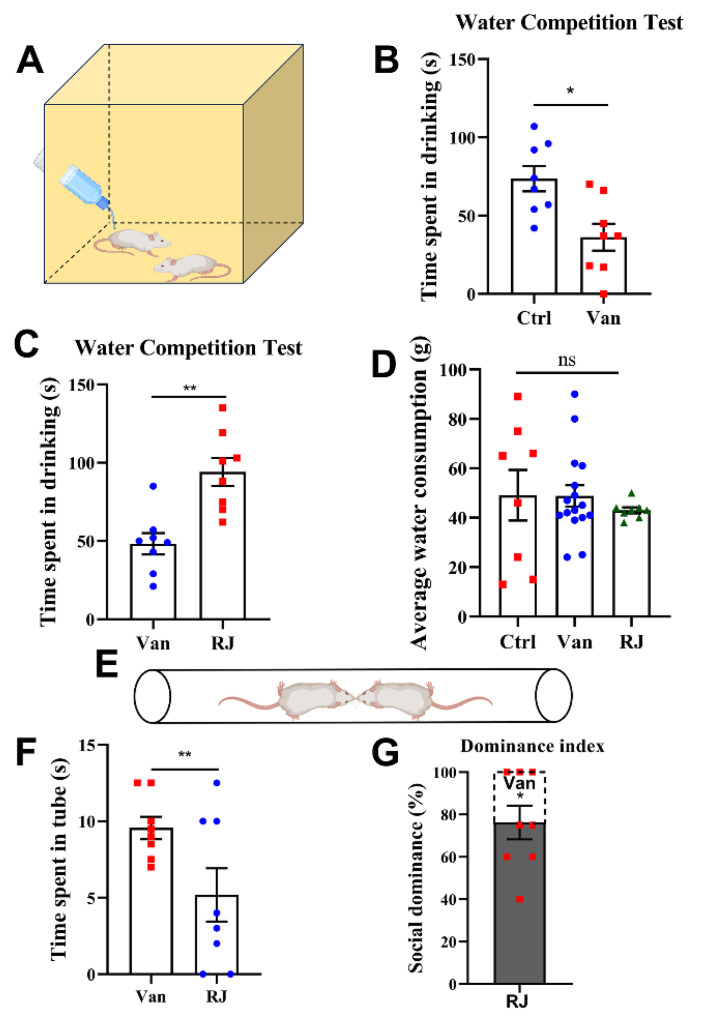
Water and tube competition tests in rats treated with vancomycin and royal jelly. (**A**) The schematic illustration of the water competition trials, where the rat pairs were placed in a new arena with a specific set of a waterspout. The social ranks were then determined by the duration each individual animal occupied (*n* = 8). (**B**) The statistical comparisons of durations occupied in the waterspout during the competitions between untreated and van-treated groups (F(7,7) = 1.13). (**C**) The statistical comparisons of durations occupied in the waterspout during the competitions between van-treated and RJ-treated groups (F(7,7) = 1.71). (**D**) The average water consumed during the test among groups. (**E**) The schematic illustration of the tube trials, where the rat pairs were placed in either entrance of a narrow tube, and their performance in this strict encounter was then observed. The social ranks were then manifested by the “advance or retreat” of an individual rat, as well as the time used to win a duel. (**F**) The time spent in the tube test of individual rats. The combat was carried out between van-treated and RJ-treated groups. The time of a winner was detracted by the difference from a 15 s threshold (F(7,7) = 5.72). (**G**) Dominance index in the tube test. This parameter was calculated based on the assigned values: winners who used less time than “Mean − SEM” in all tests, within the range of “Mean ± SEM”, or over “Mean + SEM” but less than 15 s were designated as 100, 75 and 60, respectively. The contestants that did not yield a result within 15 s were designated as 50. Statistical analysis was performed using one-way ANOVA, paired *t*-test or one-sample *t*-test with the reference variable of 50. All data are expressed as mean ± SEM from three experiments. * *p* < 0.05; ** *p* < 0.01; ns, *p* > 0.05. Ctrl, untreated group; Van, vancomycin-treated group; RJ, royal jelly and vancomycin treated group.

Given that social rank is a manifestation of complex behaviors, it is normally evaluated using multiple behavioral paradigms to ensure reliability [29]. Thus, another trial, a water competition test (Figure 2A), was also used to investigate the social activity of RJ. When it omes to the duration occupied in the waterspout, the van-treated rats displayed a significant disadvantage over their control counterparts (Figure 2B). This trend was then reversed by RJ intake during PNW6 and 7 (Figure 2C), which substantiates the rank-ascending effect of RJ. Besides, the groups did not show remarkable differences in total water consumed throughout the test (Figure 2D). This means that the variations in the waterspout occupation should not be ascribed to the extent of thirst but a genuine reflection of competition status.

This phenomenon was further validated by a third behavioral paradigm, the tube test (Figure 2E). During this test, the two rats were placed into a narrow tube, wherein the social rank was literally exhibited by the “advance or retreat” performance. As revealed by this test, RJ indeed rescued the competitive deficits of the dysbiotic rats, reaching a dominance of 76.25 ± 7.95.

Collectively, RJ improves social rank in SD rats.

### 3.2. RJ Ameliorates the Gut Dysbiosis Accompanied by Social Subordination

**Figure 3:**
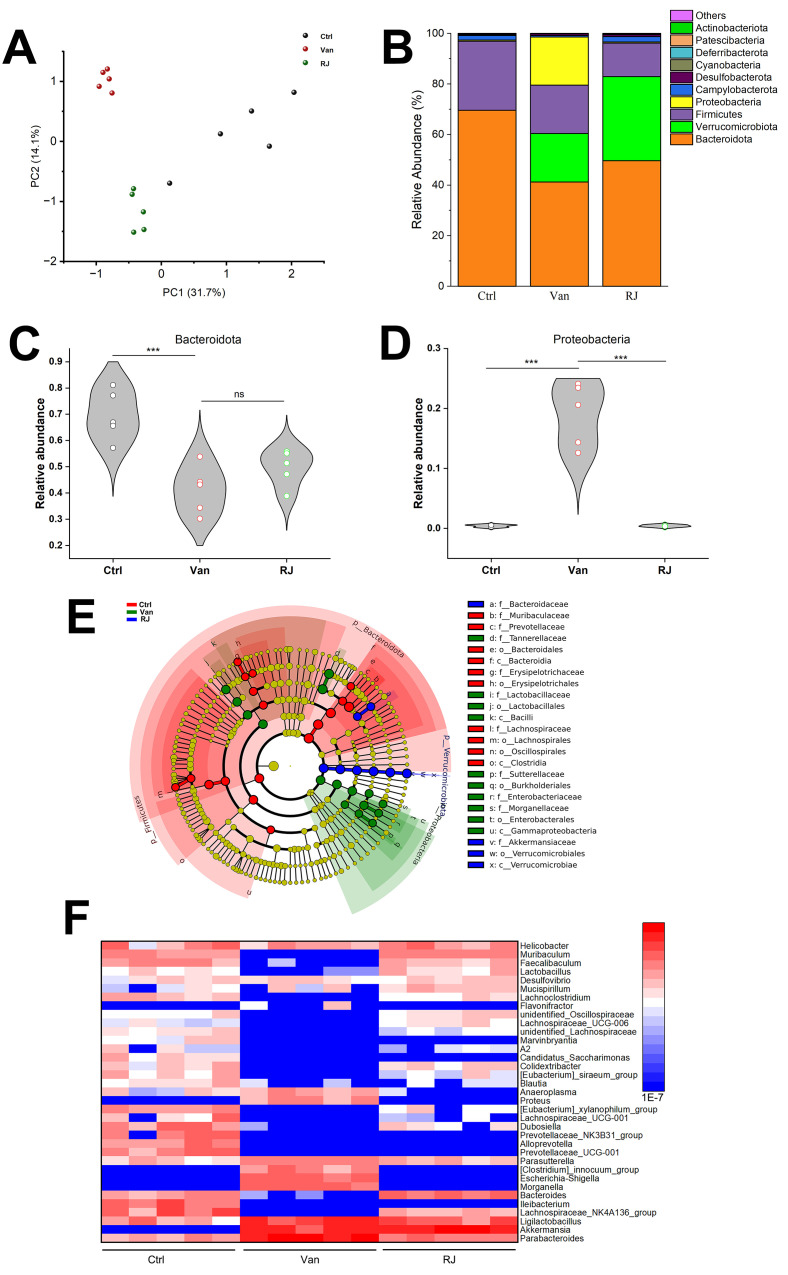
Microbiota compositions in the van and RJ-treated rats. (**A**) PCA analysis of the gut microbiota in the intestines of rats with van and RJ treatments. (**B**) Stacked compositions of various phyla harbored by the respective microbiota. (**C**) Relative abundance of Bacteroidota among treatments. (**D**) Relative abundance of Proteobacteria among treatments. (**E**) LEfSe analysis of the prevailing bacterial taxa among groups. (**F**) Heatmaps of the relative abundance of the representative genera among treatments. Statistical analysis was performed using one-way ANOVA. All data are expressed as mean ± SEM from three experiments. *** *p* < 0.001; ns, *p* > 0.05. Ctrl, untreated group; Van, vancomycin-treated group; RJ, royal jelly and vancomycin treated group.

Gut microbiota has a profound impact on social dominance, while gut dysbiosis normally results in social subordination [18]. In order to figure out the influence of RJ on gut microbiota, 16S rRNA sequencing was conducted. As shown in the PCA (principal component analysis) graph, the treatment of vancomycin caused a drastic microbial perturbation in rat intestines, which was partially reversed by the use of RJ (Figure 3A). For the specific microbial composition, it seems that the alterations of Bacteroidota and Proteobacteria were considerable among treatments, highlighted by an increase in Bacteroidota and a decrease in Proteobacteria in response to RJ (Figure 3B). However, only the RJ-triggered enrichment of Proteobacteria is statistically significant, compared to the case in Bacteroidota (Figure 3C,D). LEfSe analysis showed that the relative abundance of Enterobacteriaceae, Enterobacterales and Gammaproteobacteria prevailed among all the three treatment groups, while the addition of RJ led to the prominent existence of Akkermansiaceae and Verrocomicrobiae (Figure 3E). Based on the common knowledge on these bacteria, it might be deduced that RJ improved the microbial ecology previously disrupted by antibiotics. Regarding the genus level, the socially submissive rats harbored the increased *Clostridium*, *Escherichia* and *Morganella* compared to either untreated or RJ-treated conspecifics, implying their relevance with the studied rank variations (Figure 3F). Additionally, the use of RJ stimulated the growth of a collection of beneficial microbes in rat intestines, such as *Muribaculum*, *Lactobacillus*, *Akkermansia*, etc. Taken together, RJ remedies dysbiosis associated with social submission by maintaining a balanced microecology.

### 3.3. RJ Modulates Gene Expression in Prefrontal Context in the Genomic Scale

**Figure 4:**
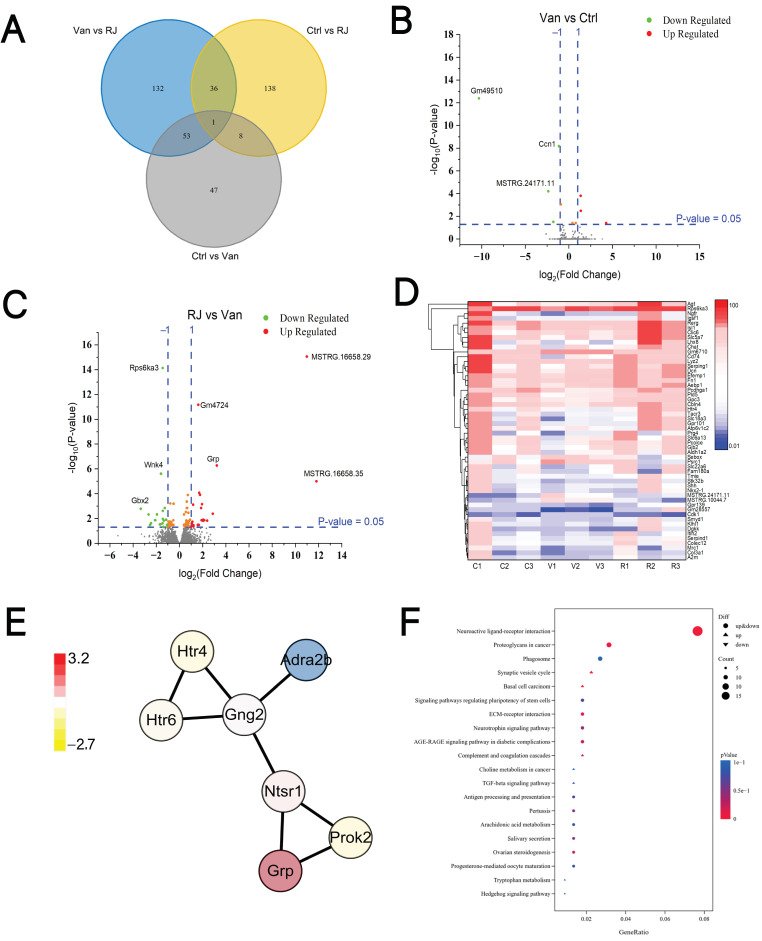
Transcriptomic analysis in the prefrontal cortex of rats treated with vancomycin and royal jelly. (**A**) Venn diagram of the number of DEGs (differentially expressed genes) among different groups. (**B**) Volcano graphs depicting the DEGs in the van-treated rats relative to the untreated controls. (**C**) Volcano graphs depicting the DEGs in the RJ-treated rats relative to the van-treated conspecifics. (**D**) Heatmap showing the expressional levels of the representative genes in the rats’ prefrontal cortex across treatments. (**E**) The interactive network of proteins upon the administration of RJ. This network was created by STRING, and the color of each node refers to the expressional changes relative to the van-treated group. (**F**) The KEGG pathways associated with differentially expressed genes (DEGs) in the settings of RJ versus van. Ctrl, untreated group; Van, vancomycin-treated group; RJ, royal jelly and vancomycin treated group.

As a brain-related behavior, social hierarchy is governed by synaptic strength in the medial prefrontal cortex (mPFC) [30]. Based on the existence of the microbiota–gut–brain axis, the cerebral gene expression is prone to the influence of gut microbiota [18,27,31]. Therefore, it is necessary to decipher the gene expression profiles in response to RJ intake, which brought significant changes in social rank (Figure 1 and Figure 2). As unraveled by the transcriptomic data, 109 genes were differentially expressed when vancomycin was used to induce dysbiosis and social subordination. In contrast with this group, the expression of 222 genes was significantly altered due to the RJ supplement, while 54 genes were commonly deregulated by both treatments (Figure 4A). When it comes to dysbiosis-related subordinance, a series of genes were upregulated, including *Grp* (gastrin releasing peptide), while *Rps6ka3*, *Wnk4* and *Gbx2* were among those downregulated during intervention (Figure 4B). When it comes to the RJ-mediated dominance, the dominant rats exhibited the suppressed mRNA levels of *Gm49510*, *Ccn1* and *MSTRG.24171.11* compared to their rivals (Figure 4C).

Heatmap shows that a subset of genes is susceptible to both vancomycin and royal jelly, displaying a reversal trend among treatments (Figure 4D). These genes might form a variety of interactive networks, represented by the reciprocal interaction of Htr4, Htr6, Gng2, Adra2b, Ntsr1, Grp and Prok2 (Figure 4E). This network was proposed by STRING, and their functional annotations suggested a close relationship with the protein signaling and synaptic transmission. Moreover, when KEGG was used to summarize signaling pathways assignable to the differentially expressed genes (DEGs) in the settings of RJ versus van, neuroactive ligand–receptor interaction was utmost highlighted due to the gene number and statistical significance (Figure 4F). Besides, multiple nerve-related pathways were also involved, manifested by the synaptic vesicle cycle, the signaling pathways regulating the pluripotency of stem cells and the neurotrophin signaling pathway.

In summary, RJ drastically remodels the gene expression in the prefrontal cortex of submissive rats.

### 3.4. Escherichia Is Correlated with Gene Expression in a Specific Module

**Figure 5:**
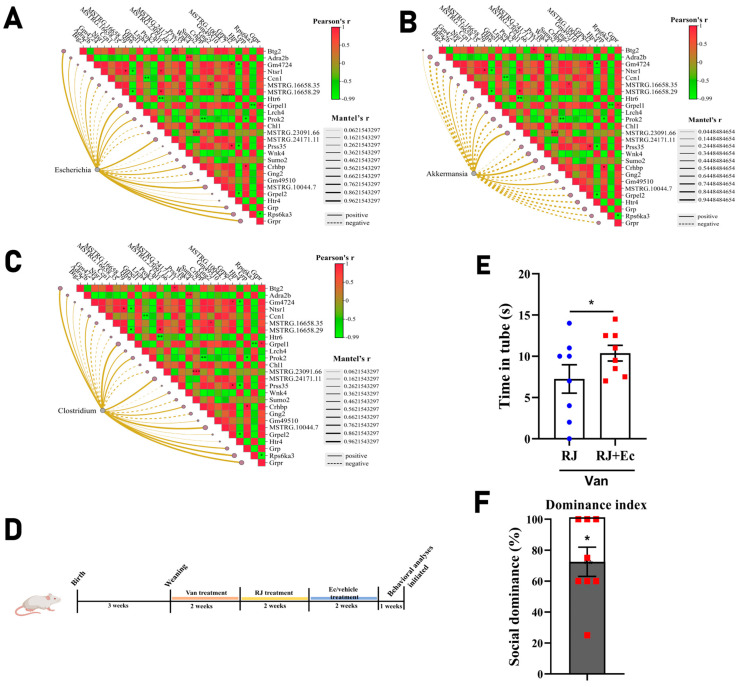
Mantel analysis of microbes with the co-correlated expression of genes in mPFC. (**A**) Mantel analysis of *Escherichia* with the gene module in rat mPFC. The Pearson’s r values between genes were denoted by the variable colors, while their correlational significance was marked by stars. The Mantel’s r value between *Escherichia* and the specific gene was expressed by the thickness of the connected lines, whereas the solid and dashed lines were used to indicate the positive and negative correlation, respectively. (**B**) Mantel analysis of *Akkermansia* with the gene module in rat mPFC. (**C**) Mantel analysis of *Clostridium* with the gene module in rat mPFC. (**D**) The experimental timeline of *Escherichia* infection in rats. Vancomycin was gavaged to male rats at a dose of 100 mg/kg at PNW4 and 5, whereas royal jelly was then added daily at a dose of 2.5 g/kg at PNW6 and 7. Subsequently, *E. coli* was colonized in the rats at a dose of 10^9^ CFU/d/rat and lasted for two weeks. The tube test was then initiated at PNW11 to evaluate the dominant status (*n* = 8). (**E**) The time spent in the tube test for individual rats. The combat was carried out between RJ-treated and Ec-treated groups. The time of a winner was detracted by the difference from a 15 s threshold (F(7,7) = 3.29). (**F**) Dominance index in the tube test. This parameter was calculated based on the assigned values: the winners using time less than “Mean − SEM” of all tests, within the range of “Mean ± SEM”, over “Mean + SEM” but less than 15 s were designated as 100, 75 and 60, respectively. The contestants that did not yield a result within 15 s were designated as 50. * *p* < 0.05; ** *p* < 0.01; *** *p* < 0.001. RJ, royal jelly-treated group; Ec, *E. coli*-treated group. Neuron-related genes are responsive to Escherichia and mediate the spine dynamics.

Next, we tried to investigate if the microbe was correlated with prefrontal gene expression in the context of RJ intervention. A multi-omics analysis was then carried out to sort out their relationship. As shown by the results (Figure 5A), *Escherichia* displayed gene-dependent associations with the prominent DEGs in response to RJ treatment. Of note, a set of genes appear to correlate with each other significantly, which expression was positively linked with the relative abundance of *Escherichia* in the gut. The most enriched correlation was represented by Grp-related genes, like *Grpr* and *Grpel*. In addition, *Akkermansia* and *Clostridium* also showed distinctive relations with the specific gene forms, while in a fundamentally divergent manner (Figure 5B,C).

To empirically study this microbe–host interplay, we further colonized the RJ-treated rats with *Escherichia coli* (*E. coli*) from PNW8 to 9 (Figure 5D). The tube test was then performed one week later to assess its influence on the social hierarchy. It was observed from behavioral trials that the introduction of *E. coli* reproduced the damage in the social rank; that is, the social dominance recovered by RJ was further degraded to subordination by *E. coli* (Figure 5E,F). Given that *E. coli* was actually inhibited by RJ in the gut (Figure 3F), it can then be concluded that RJ improved the social rank by, to some extent, moderating the abundance of *E. coli*. 

Taken together, the interplay between *E. coli* is tightly associated with the gene network and essentially implicated in the RJ-mediated rank switching.

**Figure 6:**
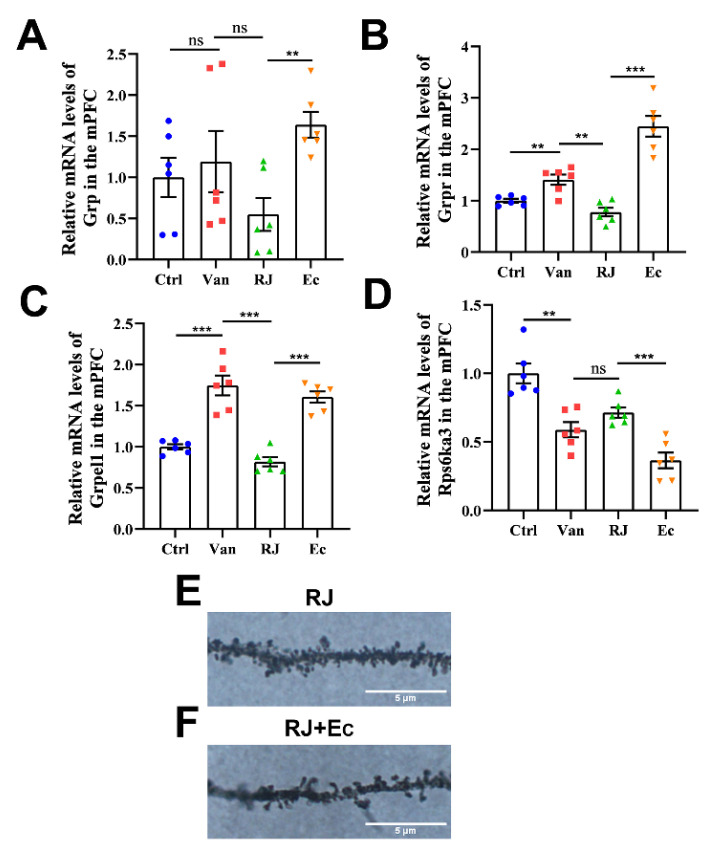
Transcript levels of specific genes in response to *Escherichia* treatment. (**A**–**D**) qPCR analysis of *Grp* (**A**), *Grpr* (**B**), *Grpel1* (**C**) and *Rps6ka3* (**D**) in response to vancomycin, royal jelly and *Escherichia* treatment. (**E**) Golgi–Cox staining was then carried out to detect the spine dynamics in the mPFC of the RJ-treated rats. (**F**) Golgi–Cox staining was then carried out to detect the spine dynamics in the mPFC of the Ec-treated rats. Statistical analysis was performed using one-way ANOVA. All data are expressed as mean ± SEM from three experiments. ** *p* < 0.01; *** *p* < 0.001; ns, *p* > 0.05. Ctrl, untreated group; Van, vancomycin-treated group; RJ, royal jelly and vancomycin treated group; Ec, *Escherichia coli*-treated group.

In the central nervous system, a gene cluster around *Grp* encodes functions related to protein signaling with emotional responses, social interaction and memory [32], while *Rps6ka3* is associated with learning and long-term memory formation [33]. To investigate whether their expressions in brains are genuinely affected by *Escherichia* in intestines, their transcript levels were further examined by qPCR analysis. Based on our findings (Figure 6A–D), *Grpr* and *Grpel* showed consistent changes with the transcriptomic data upon the supplementation of van and RJ. When *Escherichia* was introduced, the expression of both genes was significantly promoted, displaying an inverse relationship with the RJ-treated group. Besides, the level of *Grp* and *Rps6ka* was also affected by the varying manipulations, albeit with no significant differences. This result empirically validated that these genes are mechanistically responsive to the gut microbial status featuring *E. coli*.

Based on the possible function of these genes, we further inspected the spine densities in mPFC, as this is a paradigm parameter to manifest the excitatory synaptic transmission and neuronal state [34]. As shown by Golgi–Cox staining (Figure 6E,F), the spine densities were markedly weakened by the invasion of *Escherichia*. This suggests that the neuronal signaling is influenced by the microbiota status, which might have an impact on the subsequent social status.

In summary, *Escherichia* affected the neuron-related genes and modulated the spine dynamics.

## 4. Discussion

In this study, royal jelly was shown to reshape social hierarchy in rats, with gut–brain mechanisms further elucidated. Specifically, in our context, the administration of royal jelly was capable of restructuring the gut microbiota in the submissive rats, which, as represented by a decrease in *Escherichia*, in turn imposed influence on the prefrontal expression of *Grpr* and *Grpel1*. This pathway accounts for changes in neuronal transmission and the resulting social rank. This serves as an intriguing example that RJ can still modulate social hierarchy beyond honeybees or insects, suggesting that it may not just be a factor in caste determination among lower-grade animals. Given the unique status of RJ in the nutritional determinants of social behaviors (caused by dysbiosis), this research could expand our current understanding of this specific nutritional domain. Still, it needs to be noted that other factors might be implicated in the social switch in addition to royal jelly. The current finding is comparable to a series of recent studies that microbiota has an impact on neurobehavior, such as *Lactobacillus reuteri* significantly improving sociability and a preference for social novelty in maternal high-fat-diet-treated offspring by restoring the number of oxytocin immunoreactive neurons in brains [17] and indole-3-carboxaldehyde produced by *Parabacteroides distasonis* regulating depressive-like behavior in Gpr35-deficient mice by modulating neuroplasticity in the nucleus accumbens [35].

The rank-ascending effects of RJ are triggered in the submissive organisms induced by dysbiosis. This finding aligns with our prior study that the antibiotic treatment could lead to a markedly lower social rank, which can be improved by microbiome-based reprogramming [18]. Our current research proposes that RJ is able to improve social subordination by modifying gut microbial compositions. It is intriguing to notice that the supplementation of *Clostridium butyricum* can elevate the social dominance from 11.16 ± 1.34 to 44.88 ± 6.6 [18], which is inferior to the performance of RJ-mediated rescue (76.25 ± 7.95). Therefore, the notion is herein supported that the microbiome is only responsible for a fraction of dominance switching, particularly in elevating the lowest rank in dysbiotic organisms. Intriguingly, while RJ is capable of altering the gut microbiota in healthy mice [2], it did not significantly reshape the social status of untreated rats. This might be attributed to the complexity of social hierarchies, influenced by numerous interacting factors. Hence, the outcome of competitive engagement varies depending on the physiological settings of the competing individuals.

Since social hierarchy is closely linked with the mental well-being of animals and humans, it is intriguing to speculate that the current finding might be used to broaden the mental application of RJ in the fields of functional foods. Based on the emerging findings, RJ is able to exert a neuroprotective effect on human health, particularly concerning various neurodegenerative diseases [15,36,37,38]. The vast majority of these merits were mediated by the well-established antioxidative properties of RJ, but this pathway might not be significantly involved in the current context, as there was no substantial evidence supporting the antioxidative signaling pathways in the prefrontal cortex (Figure 4E). Concomitantly, social defeat is commonly associated with the occurrence of mental disorders such as depression and anxiety rather than neurodegeneration. Therefore, the proposed functions and mediatory routes in this study are likely translatable to nutritional and preclinical practices aimed at addressing psychological abnormalities.

The oral intake of royal jelly inhibits the growth of multiple pathogens within the phylum of Proteobacteria, exemplified by *Escherichia* and *Clostridium*. This result is consistent with the widely accepted knowledge that RJ has antibacterial activity [39,40]. However, this activity does not apply uniformly across all bacterial species, as some microbes showed enhanced presence in intestines following RJ treatment, such as *Akkermansia* and *Lactobacillus* (Figure 3F). In light of their probiotic status, their stimulation is supposed to constitute the beneficial jigsaw of RJ on gut microbiome and social rank. Therefore, the overall modulation should be viewed as the determinant of RJ’s effects, rather than focusing solely on its antimicrobial properties. Recent research has revealed horizontal RNA transfer from RJ to host cells, along with the identification of a diverse array of fungi and bacteria through sequencing data [41]. This study unveils the existence of a complicated consortium of microbes in this nutritional secretion. Based on this observation, these microbes might interact with the host microbiota when RJ is administered, contributing to the reshaping of the resulting microecosystem. The extent to which this interplay elicits impact on the ensuing social behaviors (caused by dysbiosis) will need to be clarified in future investigations. In addition, the usage of metabolomic sequencing will help in validating and advancing the proposed host–microbe interactions in the studies to follow.

Grp serves as an important type of neuropeptide involved in a range of neuroactive processes. It acts by binding to the GRP receptor (GRPR), activating GRPR-related signaling, which ultimately governs stress responses, fearful memory associated with synaptic plasticity, social interaction, itch-specific responses and satiety [42,43,44,45,46]. The gene cluster involved in this signaling cascade was implicated in the social-rank orientation, as RJ notably modified the expression of this functional cluster in the prefrontal cortex. Furthermore, its level was closely correlated with the relative abundance of *Escherichia* and prone to changes in the colonization of this microbe, proof that this cluster might serve as a key regulator in the studied gut–brain communications. While this prominent mechanistic relation was initially identified to interpret the nutritional intervention of brain-related behaviors, the missing link, that is, the exact messengers conveying information to the brain region, remains largely unknown. However, the initiation of these messengers is likely ascribed to the action of key metabolites produced by *Escherichia* or by cross-feeding strains. Furthermore, the roles of neuroplasticity should be better clarified by the introduction of functional experimental validations such as electrophysiology and immunohistochemistry studies. Due to the complexity of the gut–brain axis, a clear causal relationship between *E. coli* and gene expression needs to be further investigated by using germ-free mice and single-strain colonization. Another important issue is how Grp was regulated in the studied context, wherein the roles of histone acetylation might be correlated. Some studies have suggested that royal jelly can serve as an HDAC inhibitor (HDACi) [5]. On the other hand, histone acetylation has a broad-ranging effect on gene expression in multiple tissues including brains, rendering it a competent candidate serving as molecular switcher. This hypothesis warrants testing in the future.

## 5. Conclusions

In conclusion, royal jelly has been shown to effectively improve social hierarchy in submissive rats. This dominance-modulating effect was achieved by the RJ-modifying microbiota, as well as the gut–brain axis centering at *Escherichia*-Grp-related gene clusters. This study provides compelling evidence for expanding the caste-modulatory activity of royal jelly to mammals (rats), offering a promising perspective for utilizing this unique nutritional substance to support and maintain the mental well-being in other higher organisms. Furthermore, different probiotic manipulations, as well as the usage of various social models, might facilitate the uncovering of the in-depth mechanistic insight in the studies to follow. Due to social hierarchy being closely linked with mental wellness, the current finding might help us seek the translatable application of royal jelly in humans, which potentially reduces the risk of various psychiatric disorders.

## Figures and Tables

**Table 1:** Primers used in this study.

Primers	Sequences (5′-3′)	Genes
HDAC2-F	AACTTGCCGTTGCTGATGC	*HDAC2*
HDAC2-R	GCATGTGGTAACATTCGCAGA	
Grpr-F	GTGGACCCTTTCCTGTCCTG	*Grpr*
Grpr-R	GGACTTGACCGTGCAGAAGA	
Rps6ka3-F	ATGGATGAACCTATGGGAGAGG	*Rps6ka3*
Rps6ka3-R	CCAAATGATCCCTGCCCTAATAC	
Grp-F	CTGTTGGCTCTGGTCCTCTG	*Grp*
Grp-R	CATACAGGGACGGGGATTCA	
Htr4-F	GATGCTAATGTGAGTTCCAACGA	*Htr4*
Htr4-R	CAGCAGGTTGCCCAAGATG	
Grpel1-F	TTGGCACTGTCGTTCAGGC	*Grpel1*
Grpel1-R	GGATCTGTCTTTGGCTCACAAT	
GAPDH-F	TGCACCACCAACTGCTTAG	*GAPDH*
GAPDH-R	GATGCAGGGATGATGTTC	

## Data Availability

The original contributions presented in this study are included in the article. Further inquiries can be directed to the corresponding authors.
